# Update on Pharmaceutical and Minimally Invasive Management Strategies for Chronic Obstructive Pulmonary Disease

**DOI:** 10.1155/2011/257496

**Published:** 2011-04-20

**Authors:** Rokhsara Rafii, Timothy E. Albertson, Samuel Louie, Andrew L. Chan

**Affiliations:** ^1^Division of Pulmonary, Critical Care and Sleep Medicine, UC Davis School of Medicine and VA Northern California Health Care System, 4150 V Street, Suite 3400, Sacramento, CA 95817, USA; ^2^Division of Pulmonary, Critical Care and Sleep Medicine, UC Davis School of Medicine, 4150 V Street, Suite 3400, Sacramento, CA 95817, USA

## Abstract

Chronic obstructive pulmonary disease (COPD) is a debilitating pulmonary disorder with systemic effects, and it is the fourth leading cause of death in the United States. COPD patients not only develop respiratory limitations, but can also demonstrate systemic wasting, features of depression, and can succumb to social isolation. Smoking cessation is crucial, and pharmacotherapy with bronchodilators is helpful in symptom management. Inhaled corticosteroids may be beneficial in some patients. In addition, pulmonary rehabilitation and palliative care are important components under the right clinical circumstance. This review highlights current guidelines and management strategies for COPD and emphasizes novel pharmacotherapy and minimally invasive (nonsurgical) lung-volume reduction interventions that may prove to be of significant benefit in the future.

## 1. Epidemiology

Chronic obstructive pulmonary disease (COPD) is a syndrome characterized by chronic and progressive airflow reduction that is scarcely reversible and by inflammation of the small airways. It is the potential functional consequence of two diseases that can often coexist in the same patient, such as panlobular emphysema and fibrosing chronic bronchiolitis with or without significant centrilobular emphysema. It can also include chronic bronchitis (the presence of a chronic productive cough for 3 months or more in each of 2 consecutive years) [1, 2]. Chronic bronchitis per se is a smoking related disease of large airways that often resolves after smoking cessation. Nevertheless, patients with COPD who suffer from chronic bronchitis generally show faster functional decline, more exacerbations, and greater morbidity and mortality. Furthermore, a greater percentage of subjects with chronic cough and phlegm who continue to smoke can have COPD as compared with smokers without symptoms when functionally reassessed after 8 years [3]. However, the majority of patients with chronic bronchitis will not suffer from COPD [2, 3]. Therefore, chronic bronchitis itself can be considered as both a risk factor for COPD, and a worse prognostic factor in the presence of COPD.

COPD typically progresses over time and is associated with an increased inflammatory response of the lung to continued environmental exposures which is often tobacco smoke [4]. The natural history of COPD is punctuated by breathlessness especially on exertion with daily activities of normal living, increased production and purulence of sputum, overall health decline, and episodes of exacerbations that require medical attention and hospitalizations. 

While the prevalence of COPD varies by country, it is generally linked to the prevalence of tobacco smoking. There is also a link to air pollution from the burning of wood and other biomass fuels [4]. The prevalence of chronic bronchitis among adults from 1999–2008 ranged from 34 (2007) to 55 (2001) cases per 1,000 population in the United States (USA). The range over the same time period for emphysema was 14 (1999) to 18 (2006) cases per 1,000 population [5]. In 2008, females had twice the reported prevalence of chronic bronchitis than males (58 versus 29 cases per 1,000 resp.). Emphysematous males have a slightly higher prevalence than females (17 compared to 16 cases per 1,000, resp.) [5]. Gender differences may separate clinical COPD phenotypes and is typical of the heterogeneity in COPD. 

Worldwide, COPD is one of the leading cause of morbidity and mortality [4]. COPD is the 4th leading cause of mortality in the USA, and is also the only one of the top five leading causes of death that is continuing to rise, doubling from 1970 to 2002 [6]. It is projected that COPD will become the third leading cause of death worldwide by 2020 [4]. Furthermore, COPD deaths among women in the USA have been rapidly rising since the 1970s and have exceeded male COPD deaths since 2000 [4, 7]. 

COPD presents an increasing social and economic burden. COPD patients incur health care costs associated with frequent clinic visits, urgent care visits, and hospitalizations. Home medical therapies, including oxygen therapy, visiting nursing services, and rehabilitation add to the cost [4]. The health-care expenditure for each COPD patient cost on average $6,000 annually [8]. In 2002, the estimated USA direct medical cost of COPD was $18 billion while indirect costs including lost wages and decreased productivity were estimated at $14.1 billion [4]. 

## 2. Current Treatment Guidelines

The goals of COPD treatment are to arrest or at least reduce its progression, control symptoms, and to prevent acute COPD exacerbations in an attempt to improve overall mortality. Smoking cessation, pharmacotherapy, and pulmonary rehabilitation form the cornerstones of COPD management.

### 2.1. Smoking Cessation

Smoking cessation programs and education should be available and encouraged for all smokers. The Global Initiative for Chronic Obstructive Lung Disease (GOLD) guidelines emphasize that smoking cessation is “the single most effective and cost-effective way to reduce exposure to COPD risk factors [4].” In a randomized, controlled trial of a 10-week-long smoking cessation program in 5887 smokers with asymptomatic mild-moderate airway obstruction, the 14-year all-cause mortality rates in the smoking cessation group was lower than in the usual care group (HR 1.18; CI 1.02–1.37) [9]. In addition to counseling, a variety of effective pharmacotherapies for smoking cessation are available for patients, the details of which are beyond the scope of this review. 

### 2.2. Pharmacotherapy

GOLD has classified COPD into four stages based on spirometric values and thus on severity of airflow obstruction (Table 1) [4]. The focus of COPD treatment should be relief of symptoms, improving exercise tolerance and overall health status, prevention and timely treatment of exacerbations, preventing disease progression, and reducing mortality [4]. Pharmacologic therapy is able to significantly improve the quality of life and reduce the frequency of exacerbations, but is unable to halt the annual decline in FEV_1_ or unequivocally reduce mortality, with the important exception of oxygen therapy where indicated [10]. 

Bronchodilators are the mainstay for the symptomatic management of COPD. While they do not alter the decline in lung function, they decrease expiratory trapped air volume and reduce dynamic hyperinflation during exercise as well as (in the more severe cases) at rest [4]. They are prescribed in both short- and long-acting forms for immediate (rescue) and sustained relief, respectively.

Long-acting bronchodilators are recommended for patients with moderate to severe COPD. In the largest trial of a salmeterol, long-acting beta-2 agonist (LABA), Toward a Revolution in COPD Health (TORCH), patients with a mean forced expiratory volume in one second (FEV_1_) of 44% of predicted (thus mostly severe COPD) were randomly assigned to one of four treatment arms for 3 years: salmeterol alone (50 mcg twice daily), fluticasone alone (500 mcg twice daily), the combination of both, versus placebo [11]. The salmeterol alone and combination arms each revealed improved lung function, health-related quality of life, and reduced exacerbation rates compared to the placebo arm. No statistically significant mortality reduction was seen. However, the combination of fluticasone and salmeterol showed a trend towards a significant (*P* = .052) reduction of all-cause mortality by 17.5% in 3 years compared to placebo. 

The effects of the long-acting anticholinergic, tiotropium, was assessed in a randomized trial Understanding Potential Long-Term Impacts on Function with Tiotropium (UPLIFT) [12]. Among patients with moderate (45%) and severe (44%) COPD, tiotropium or placebo was added to ongoing care, that is, inhaled corticosteroids, long-acting beta-2 agonists, and/or theophylline, for a duration of 4 years. Tiotropium significantly reduced the risk of exacerbations and associated hospitalizations and respiratory failure when added to usual care, while there was no difference in the annual rate of decline of FEV_1_ or mortality between the two groups [12]. However, in an intention-to-treat analysis that assessed mortality after 4 years, the use of tiotropium reduced all cause mortality by 13% (HR = 0.87, 95% CI 0.76–0.99, *P* < .034) [13]. 

The combination of bronchodilators of different classes and durations may provide an improved effect with fewer side effects [4]. For example, the combination of a LABA and an anticholinergic (tiotropium) showed an improved FEV_1_ by the end of a 12-week trial [14]. In another study, the use of a short acting beta-agonist plus an anticholinergic showed a greater and longer-lasting FEV_1_ improvement when compared to each drug alone [15, 16]. However, no clear data are available as to which long-acting bronchodilator combination provides the best relief. Current guidelines recommend weighing the risks and benefits of each for the individual patient [4].

Although pulmonary inflammation plays an important role in the pathophysiology of the disease, inhaled corticosteroids (ICS) have not definitively been shown to decrease the rate of lung function decline or change COPD morbidity per se. However, the addition of an ICS may prove beneficial for some. In the TORCH study above, the combination of a LABA plus ICS resulted in a decrease in the number of exacerbations along with sustained benefits in health status and sustained improvement in FEV_1_ when compared to placebo, salmeterol alone and fluticasone alone [11]. This combination is typically reserved for patients with GOLD stages 3 and 4 with recurrent exacerbations, or for those who have baseline eosinophilic component of their disease or associated asthma. 

Recently, this relationship of airway hyperresponsiveness and the long-term effects of ICS were also investigated in a smaller randomized controlled trial of 114 patients with moderate to severe COPD who had not used ICS for at least 6 months [17]. Patients were randomized into four treatment arms: fluticasone for 6 months followed by 24 months of placebo, fluticasone for 30 months, fluticasone and salmeterol for 30 months, or placebo only. Those treated with placebo or with fluticasone for the first 6 months had a decline in FEV_1_ of −87 mL/year, and −65 mL/year respectively. Those treated with fluticasone for 30 months or fluticasone and salmeterol had a significantly reduced rate of FEV_1_ decline (+7.3 mL/year for the former and −16 mL/year for the latter). Overall, fluticasone significantly diminished annual FEV_1_ decline over the last 2 years of the study when compared with placebo (CI, 43 to 129 mL/year; *P* < .001). It is important to note that 95% of these patients had never used ICS, and the majority of patients had airway hyperresponsiveness and revealed some acute reversibility of FEV_1_. Thus, this study may be representing a subgroup of COPD patients who may respond favorably to ICS [18]. 

A recent meta-analysis readdressed the possibility of a favorable effect on mortality when combining ICS and bronchodilators in COPD [19]. Use of an ICS combined with LABA demonstrated a 20% reduction in total mortality, with a mortality risk ratio of 0.80 (95% CI, 0.69–0.94) [19]. However, use of a LABA or tiotropium alone did not decrease the mortality rate [19]. There is also concern that the use of high dose ICS in patients with COPD may be associated with an increased risk of pneumonia. The risks and benefits of these medications must be weighed for each patient [20, 21].

### 2.3. Nonpharmacologic Interventions: Pulmonary Rehabilitation and Palliative Care

In order to effectively treat patients with COPD, it is important to understand the physical decline, increased sense of isolation, changes in mood such as depression, muscle wasting, and weight loss that can afflict such patients [4]. Thus, comprehensive therapy must include promoting a healthy life style including smoking cessation, appropriate vaccinations, and encouragement to remain physically active [22]. 

 Pulmonary rehabilitation is designed to “reduce symptoms, optimize functional status, increase participation, and reduce health care costs through stabilizing or reversing systemic manifestations of the disease [23].” Data from clinical trials reflect that pulmonary rehabilitation increases peak workload by 18%, peak oxygen consumption by 11%, and endurance time by 87% from baseline, but do not change lung function or mortality [4]. Pulmonary rehabilitation should be considered to be complementary to pharmacological therapy and those with GOLD stages 2, 3, and 4 should be encouraged to participate [4, 22].

The integration of palliative care among patients with severe COPD is often overlooked. When compared to patients with lung cancer, patients with severe COPD receive less palliative care even though they suffer from the same symptoms of dyspnea, depression, anxiety, pain, difficulty sleeping, nutritional problems, cachexia, social isolation, and functional disability [24]. Additionally, pain is often underappreciated in severe COPD patients, despite the fact that it is a symptom that is nearly as prevalent as in patients with lung cancer (21% versus 28%, resp.) [25]. Given its multidisciplinary approach, pulmonary rehabilitation programs provide a practical venue for incorporating palliative care for these patients [24]. 

To date, the only therapy besides smoking cessation that has been shown to statistically prolong survival in patients with COPD is oxygen supplementation among those with severe hypoxemia [22]. The administration of oxygen is appropriate for COPD patients who have a PaO_2_≤ 55 mmHg or SaO_2_≤ 88%. A PaO_2_ between 55 mm Hg and 60 mm Hg or SaO2 ≥ 88% merits supplemental oxygen if the patient has pulmonary arterial hypertension, peripheral edema suggesting congestive cardiac failure, or polycythemia (hematocrit > 55%). A survival benefit was found in two small studies for patients who qualified for oxygen therapy and used it for greater than 15 hours per day [26, 27]. Nevertheless, the 2-year mortality is 49% in patients being treated with oxygen [28]. 

While the adverse health effects of COPD have impacted people and health care systems worldwide, the ability to modify or even halt disease progression remains limited. Clearly earlier COPD case findings, diagnosis, and treatment may be beneficial and has fueled the development of novel pharmacological interventions.

## 3. Novel Pharmacotherapy for COPD

Many of the current drugs used for COPD were adopted from their application in asthma [1]. However, the pathophysiology of COPD is markedly different from asthma, and the chronic inflammation seen in COPD is predominantly from an influx of neutrophils, macrophages, and CD8 (cytotoxic T-helper) lymphocytes [1]. Eosinophils are seen in lung biopsies but to a much lesser extent than in asthma. CD8 lymphocytes are found in abundance in COPD but have a different role than in asthma [29]. By releasing tumor necrosis factor-alpha (TNF*α*) and IL-12, macrophages contribute to the destruction, remodeling, and continued inflammation in the lung parenchyma. They are also a source of matrix metalloproteinases that destroy collagen and elastin [30]. The result is narrowing of small airways from peribronchial fibrosis and destruction of alveolar walls [1]. There is a need for therapies that are formulated with a better understanding of the targets specific to COPD. The following describes pharmacotherapy that is currently under study to modify COPD through mechanisms other than bronchodilation.

### 3.1. Phosphodiesterase (PDE) Inhibitors

Inflammatory cells such as neutrophils, CD8 lymphocytes, and macrophages, express predominantly phosphodiesterase (PDE) type 4 [1]. PDE type 4 hydrolyzes cyclic adenosine monophosphate (cAMP) in inflammatory cells. By inhibiting PDE type 4, intracellular cAMP concentrations increase which leads to activation of protein kinase A, phosphorylation and inactivation of target transcription factors, which ultimately result in reduction of cellular inflammatory activity [31]. 

It is predicted that PDE type 4 inhibition will provide better antiinflammatory activity in patients with COPD than corticosteroids because the latter does not suppress neutrophil activation or production of cytokines and chemokines compared to the former [31]. While theophylline is a nonspecific PDE inhibitor (thus with a large side-effect profile including diarrhea, seizures and cardiac arrhythmias), several new drugs have been tested that target PDE type 4 specifically, notably cilomilast and roflumilast. 

A dose-ranging study of the selective PDE type 4 inhibitor cilomilast in COPD patients showed significant improvements in FEV_1_, although quality of life measures were not different [32]. The safety and efficacy of cilomilast (15 mg twice daily) was evaluated in a double-blind placebo-controlled study and showed significant increase in FEV_1_ as well as fewer exacerbations over a 24-week period [33]. Gastrointestinal side effects (nausea and diarrhea) were greater in the first 3 weeks of the study in the treatment arm [33]. Cilomilast has been evaluated in three additional multicenter, randomized, placebo-controlled phase III trials. The change of FEV_1_ (from 30–40 mL) compared to placebo was significant in only two of the four studies. An increase in exacerbation-free survival and decrease of the relative risk of level 2 or 3 exacerbations were significant in only two of the four trials [34]. Overall, these studies did not show as large of improvements in FEV_1_ as was expected based on phase II trials [34]. 

Roflumilast is a more potent PDE type 4 inhibitor compared to cilomilast [35]. In a phase III multicenter placebo-controlled trial, 1411 patients were randomly assigned to receive roflumilast 250 mcg, roflumilast 500 mcg, or placebo daily for 24 weeks [31]. Postbronchodilator FEV_1_ at the end of treatment significantly improved for both groups of roflumilast when compared to placebo (74 mL with the lower dose and 97 mL with the higher dose medication). Both groups suffered fewer mild exacerbations while moderate to severe exacerbations were unchanged [31]. The most common side effects were also diarrhea and nausea. 

Two randomized clinical trials on the use of Roflumilast compared to placebo were published in 2009 [36]. Patients in these studies had COPD with severe airflow obstruction, documented cough and sputum production, and a history of frequent COPD exacerbations in the past year. In pooled analysis, the prebronchodilator FEV_1_ increased by 48 mL among those with the treatment drug, a statistically significant improvement compared to placebo [36]. Moderate to severe exacerbations were also significantly less in the treatment arm. Some of the same authors published the results of another set of trials that compared the use of roflumilast to placebo when added to either salmeterol or tiotropium [37]. When compared to placebo, the addition of roflumilast to salmeterol was associated with a 49 mL prebronchodilator FEV_1_ increase, and an 80 mL increase when added to tiotropium. Moderate to severe exacerbations were noted to be significantly less only when compared to salmeterol [37]. Side effects such as nausea, diarrhea and weight loss contributed to greater patient withdrawal [37]. While results with roflumilast are encouraging, the USA Food and Drug Administration (FDA) issued a response letter requesting further information and analyses from the manufacturers in May 2010 [38]. The drug has however been granted marketing authorization by the European Commission for several European countries since July 2010 [39].

### 3.2. N-Acetylcysteine (NAC)

NAC has indirect and direct antioxidant properties [40]. Its free thiol group reacts with radical oxygen species (ROS). The presence of ROS has been shown to decrease collagen and elastin formation as well as increased IL-1 and IL-8 production, thus enhancing inflammation [40]. NAC exerts indirect antioxidant effects as a precursor to glutathione (GSH), a tripeptide that protects against both internal toxins (agents from aerobic respiration and metabolism of phagocytes) as well as external toxins (components of cigarette smoke and pollution) [40]. Some of the oxidant-antioxidant effects of NAC in the lung include increased lung lavage levels of GSH, decreased superoxide anion production by alveolar macrophages and decrease *in vitro* adhesion of *Haemophilus influenza* and *Streptococcus pneumoniae* to oropharyngeal cells [40–43]. 

Meta-analyses have shown that oral intake of NAC decreases exacerbation in patients with chronic bronchitis [44, 45]. However, in a large, randomized placebo-controlled study, there was no improvement in lung function nor a decrease in the frequency of exacerbation with a daily oral administration of NAC (600 mg) to COPD patients [46]. The risk of exacerbations was nevertheless lower in the treatment arm among patients not taking inhaled corticosteroids (HR 0.79, 95% CI 0.631 to 0.989) [46]. This study has been criticized because of the low dose of NAC that was administered. Patients with moderate-severe COPD on 1200 mg versus 600 mg daily of NAC demonstrated a more effective normalization of C-reactive protein levels and decreased serum IL-8 levels [47]. Further trials with higher doses of NAC are needed before a conclusive recommendation of NAC therapy in COPD can be made.

### 3.3. TNF*α* Inhibitors-Infliximab

Tumor necrosis factor (TNF)*α* is an ubiquitous cytokine. Given its ability for chemotaxis and activation of macrophages and neutrophils, TNF*α* is an attractive target in the effort to decrease inflammation in COPD patients with elevated TNF*α* levels [48]. Such increased levels in advanced COPD may contribute to skeletal muscle apoptosis and thus muscle wasting [49]. 

Infliximab, the humanized monoclonal antibody directed against TNF*α*, was used in a multicenter, randomized, double-blind placebo-controlled dose-finding study. Patients with moderate-severe COPD were given infliximab (3 mg/kg or 5 mg/kg versus placebo over a 24-week period [50]. While well tolerated, there was no significant improvement in the primary endpoint of disease-related health status, or in secondary outcomes such as risk of exacerbations. Though not statistically significant, the authors found a greater number of cases of cancer and pneumonia in the treatment groups [50]. There may yet be a benefit in using this therapy in severe COPD with a wasting syndrome, but the increased number of malignancies is of concern, and further study is warranted [50].

### 3.4. ABX-IL8

The use of monoclonal antibody recognizing interleukin (IL)-8 has been assessed *in vitro *and has been hypothesized to reduce neutrophil migration and activation in the airways and lung tissue [51]. To date, there is only one randomized, double-blind placebo-controlled trial that utilized this approach in moderate-severe COPD [51]. In this study, three IV infusions of a human monoclonal IgG2 antibody directed against human IL-8 (ABX-IL8) or placebo infusion were administered over a three-month period. A statistically significant improvement in the primary outcome measure, dyspnea (as measured by the Transitional Dyspnea Index) was found in the ABX-IL8 group versus placebo [50]. However, there were no significant differences in lung function parameters, health status, or 6-minute walk distance. Adverse events were similar between the two groups, and the drug was well tolerated overall. This pilot study emphasizes the potential importance of addressing the inflammatory component of COPD.

### 3.5. Antileukotriene Drugs

Neutrophilic inflammation is likely a significant cause of mucus hypersecretion and contributes to the destruction or remodeling of the lung architecture as seen in COPD. Drugs suppressing neutrophil influx into the lungs make an attractive untapped approach to preserving lung function in COPD. High levels of leukotriene B4 (LTB4), a proinflammatory derivative of arachidonic acid, have been found in the sputum of patients with COPD [1]. LTB4 is both a chemoattractant and an activator of neutrophils [52]. 

The effects of a leukotriene synthesis inhibitor, BAYx1005, among patients with COPD were assessed in a small phase II trial [53]. After 14 days of treatment, a significantly greater median reduction in sputum LTB4 compared to placebo, though no significant difference was found in the absolute LTB4 concentrations between the two groups [53]. Inhibition of leukotriene synthesis may affect neutrophilic bronchial inflammation in patients with COPD, and further studies are warranted for this class of medications [53].

### 3.6. Prophylactic Use of Macrolides

Macrolides are now used for chronic diseases such as diffuse panbronchiolitis, noncystic fibrosis bronchiectasis, asthma, and cystic fibrosis to reduce airway inflammation [54]. Macrolides have both antibacterial and antiinflammatory activity, and the latter is evident even in low inhibitory concentration for airway bacteria suggesting that the two may have independent effects [55]. 

Over the past decade, an increasing body of evidence has suggested a beneficial role in the chronic use of macrolides for patients who suffer from frequent COPD exacerbations [56, 57]. The largest trial to date was a randomized, double-blind placebo-controlled study of erythromycin given 250 mg twice daily over a 12-month period to patients with moderate-severe COPD [55]. The treatment and placebo groups did not differ from each other with regards to stable FEV_1_, sputum IL-6, IL-8, bacterial flora, serum C-reactive protein or serum IL-6. However, there was a statistically significant reduction in exacerbations over the one-year period in the treatment group [55]. While current guidelines for the treatment of COPD do not endorse the chronic use of antibiotic therapy, an increasing number of studies support the use of macrolides at least among those with the symptoms of chronic bronchitis and an increased number of yearly COPD exacerbations.

### 3.7. Vitamin D

A link between vitamin D deficiency and several chronic illnesses such a malignancies, autoimmune diseases, infectious and cardiovascular illnesses has been suggested but remains controversial [58]. In studies examining vitamin D levels in COPD, a review of spirometric data from the NHANES III showed a positive relationship between serum levels of 25-hydroxyvitamin D (25-OHD) and pulmonary function (assessed by FEV_1_ and FVC) [59]. A cross-sectional study found that more than 50% of patients awaiting lung transplant were vitamin D deficient [60]. In light of the growing interest in this field, Janssens and colleagues measured serum 25-OHD levels in 414 patients with COPD [58]. They found that decreased 25-OHD levels correlated significantly with decreased FEV_1_. When compared to smokers with normal lung functions, 60% and 77% of patients with GOLD stage 3, and 4, respectively, were deficient in 25-OHD levels. The authors also further characterized the patients according to their vitamin D-binding gene variants and those at increased risk of vitamin D deficiency [58]. Although replacement has yet to be shown to modify COPD, the low-side-effect profile of vitamin D makes it an attractive potentially therapeutic agent for further research.

## 4. Surgical and Minimally Invasive (Nonsurgical) Approaches

### 4.1. Surgery

Lung-volume-reduction surgery (LVRS) was initially proposed as a palliative treatment for those with severe emphysema. In 2003, the National Emphysema Treatment Trial (NETT) research group evaluated the effects of LVRS with a goal of better understanding which patients would benefit from this intervention [61]. They found a survival advantage among former smokers with upper lobe predominant emphysema and low baseline exercise capacity (NETT). Exercise capacity was improved by 10 W in 28, 22, and 15% of LVRS patients at 6-, 12-, and 24-month followup respectively. This was compared to 4%, 5%, and 3% 10 W improvement in patients in the medical therapy group [61]. The LVRS group was also more likely to have improved 6-minute walk distance, FEV_1_% predicted, level of dyspnea, and disease-specific and general quality of life results compared to the medical group [61]. 

The NETT research group also analyzed the morbidity and mortality data. The incidence of overall mortality within 90 days was 7.9% (95% CI, 5.9–10.3) in the surgery group compared to 1.3% (95% CI, 0.6–2.6) in the medical therapy group (*P* < .001), and a predictor of mortality was having nonupper lobe predominant emphysema. Among patients not labeled high risk, 90-day mortality was 5.2% in the surgery group as compared to 1.5% in the medical therapy group (*P* = .001). Morbidity was higher among older patients, those with lower FEV_1_ (<20%) or lower DLCO (<20%) [61]. The rate of at least one postop complication within 30 days was 58.7%, with the most common ones being arrhythmias, pneumonias, and reintubations. Air leaks were documented in 90% of surgical patients, and this prevalence and duration of air leaks was increased by patient factors such as increased airflow obstruction, use of inhaled corticosteroids, and lower diffusion capacity [62]. Furthermore, 28.1% of patients were hospitalized, living in a nursing home or rehabilitation facility, or unavailable for interview at 1 month after LVRS [61].

### 4.2. Minimally Invasive (Nonsurgical) Approaches

Estimates of the number of patients who might benefit from LVRS or lung-volume reduction (LVR) using bronchoscopic minimally invasive approaches vary wildly from a total of 1.35 million patients to several hundred suitable patients per year [10, 63]. The cost per procedure using the latter approach is estimated at between $12,000 to $20,000 [10]. LVRS may be beneficial for a subgroup of patients with severe COPD, but its risks can significantly outweigh the benefits for a large number of the COPD population. Thus, nonsurgical minimally invasive alternatives to LVRS are being explored. The use of endobronchial blockers, bypass methods, valves and sealants will be discussed here.

### 4.3. Endobronchial Blockers (Plugs)

Mechanical blockers were used in 8 COPD patients [64]. They were designed to occlude the distal airway, resulting in distal parenchymal collapse and thus LVR. The initial blockade was performed via detachable, silicone balloons, filled with contrast material to allow for radiologic identification. However, poor performance due to migration (five episodes in 3 patients), and in one case dislodgement and expectoration of the balloon in recovery, prompted the authors to switch to stainless steel wire stents containing biocompatible sponges [64]. The increased rate of blocker migration, risk of postobstructive pneumonia, and need for repeat procedures appear to have limited further development of this modality [65].

### 4.4. Bronchial Fenestration and Airway Bypass

The mechanism underlying airway bypass entails constructing artificial pathways (fenestrations) through the bronchial wall into the surrounding parenchyma, and placing a stent to maintain patency [66]. The formation of the new conducting expiratory airways allows for trapped air to escape via a less resistant and nonobstructed path. The feasibility of this procedure was demonstrated when 12 human lungs were removed at time of transplant, placed in an airtight ventilation chamber and the bronchus attached to a pneumotachometer [67]. Using a flexible bronchoscope, a radiofrequency catheter created passages in which stents were deployed. The FEV_1_ increased from an average of 245 mL at baseline to 447 mL after the placement of three stents, and to 666 mL after placement of 5 stents, confirming feasibility of the concept [67]. The safety of the procedure was assessed in 10 patients undergoing lobectomies for neoplasm [68]. The procedure was performed after thoracotomy and immediately before resection, and entailed the use of a bronchoscope to reach the target site, a doppler probe to avoid blood vessels at the target site, and the development of passages through the bronchial wall with a cautery probe [68]. Subsequently, the authors performed the procedure in 5 patients undergoing lung transplant for emphysema. A total of 47 passages were created with 2 episodes of mild bleeding, both controlled with suction and the topical application of epinephrine [68]. 

The addition of drug-eluting stents to the fenestrations was evaluated *in vitro* [69, 70]. With the use of paclitaxel-eluting stent placement in the fenestrations among thirty-five patients with severe COPD, adverse events included pneumomediastinum in two patients and one episode of major bleeding that resulted in the death of the patient. The authors noted that this fatal hemorrhage was due to stent placement away from the original spot identified with the Doppler probe [70]. Two COPD exacerbations occurred within one month of treatment and five respiratory infections occurred in the first week after intervention. At 6-month followup, there was a statistically significant decrease in mean RV by 400 mL and level of dyspnea as measured by the modified Medical Research Council Scale. The authors found lasting benefit only in patients whose baseline RV/TLC was below the median [70]. There was no significant improvement in FEV_1_, 6-minute walk distance, or St. George's Respiratory Questionnaire (SGRQ) [70]. Only small trials thus far have been able to provide information on the safety as well as efficacy of the use of airway bypass and stents. Larger clinical trials with longer followup periods may further elucidate benefits, if any, and in the case of the latter, also help identify suitable patient subgroups most likely to benefit including those with homogenous emphysema.

### 4.5. Endobronchial Valves

Endobronchial valves (EBV) are one-way valves that prevent air from entering the airway distally but allow for ventilation of the expired gas and drainage of distal secretions [67]. To date, two-valve designs have been studied in separate multicenter reports (Zephyr EBV, Pulmonx Corp and IBV, Spiration, Inc.) [65, 71, 72]. The Zephyr EBV, formerly known as Emphasys EBV, consists of a stent-like self-expanding retainer made of nitinol, which is wrapped in molded silicone [71]. In the center of the retainer is a duckbill one-way valve that allows outflow of gas and secretions during exhalation but does not permit air entry during inhalation [10, 71]. The EBV is compressed via a loader system, placed onto a delivery catheter, and a guidewire directs this catheter to the targeted area, all via the working channel of a bronchoscope [71]. 

The Intrabronchial Valve (IBV; Spiration) is also made of nitinol and has 5 distal anchors and 6 proximal support struts that are covered by a synthetic polyurethane polymer [72]. These struts expand to form an umbrella shape to allow for sealed placement in the airway. Air and mucus are able to flow around the edges of the membrane. The valve has two delivery system options via a loading device through the working channel of a flexible bronchoscope [72]. 

Several clinical trials have added understanding of the feasibility and efficacy of endobronchial valves. In a retrospective analysis from nine centers in seven countries, a total of 98 patients with heterogeneous involvement of emphysema and average baseline values of predicted FEV_1_ of 30.1%, RV of 244.3%, TLC of 128.4%, and DLCO 32.7% were enrolled [71]. A total of 396 valves were placed, with an average per patient of 4.0 (range 1–8). Statistically significant improvements were noted in FEV_1_ (10.7 ± 26.2%, *P* = .007), FVC (9.0 ± 23.9%, *P* = .024), RV (−4.9 ± 17.4%, *P* = .025), and exercise tolerance (23.0 ± 55.3%, *P* < .001) [71]. There were 8 (8%) serious complications (3 pneumothoraces requiring surgical intervention, 4 prolonged air leaks, and one death). The patient who died developed progressive pneumonia and died from respiratory failure on postprocedure day 25 [71]. Seventeen patients had acute exacerbations of COPD. 

The EBV valve was next tested in The Endobronchial Valve for Emphysema Palliation Trial (VENT) [73]. This was a multicenter, randomized controlled trial of 321 subjects with severe heterogeneous emphysema (220 treated with Zephyr EBV and 101 treated as controls). At 6-month followup, the treatment group had statistically significant improvements in the primary endpoints of FEV_1_ (+6.8%, *P* = .002) and 6MWT (+5.8%, *P* = .019). There were also significant improvements in secondary outcomes of St. George's Respiratory Questionnaire (SGRQ) and BODE index (a measure of body mass index, degree of airflow obstruction, dyspnea, and exercise capacity) compared with control [73]. The 6-month mortality was 2.8% among the treatment group (zero in the control group), and cumulative mortality rate over 1-year followup was 3.7% for the Zephyr group and 3.5% for the control group (*P* = 1.000). Complications related to the device were valve migration, pneumonia distal to the valve, and granulation tissue. In final review of various outcomes from this trial, the FDA advisory panel recommended against approval of this device, citing that the benefits were not large enough to overcome the risks [74].

The largest trial reported to date with the Spiration IBV valves was in an open-label study [75]. A total of 98 patients were enrolled at 13 international centers over a 3-year period with the intent of bilateral treatment of upper lobe predominant emphysema. Bilateral treatment was done in 95 of the 98 patients, and a total of 659 valves were placed [75]. While they did not find statistically significant improvements in spirometry and lung volume measurements at 3 and 6 month followup, the patients SGRQ decreased by greater than 4 (a 56% improvement) at 6 months. There were a total of 8 pneumothoraces, one of which was a tension pneumothorax that occurred on postprocedure day 4 and resulted in the death of the patient [75]. There were no episodes of valve migration or expectoration. The most common postprocedure adverse event was bronchospasm, which resolved after one bronchodilator treatment in 3 cases but required several repeated treatments in 2 other patients. In the latter two cases, the bronchospasm lasted for 24 to 48 hours, resolved after valve removal, and was assumed to be related to the valves [75].

To date, trials utilizing bronchoscopic LVR do not consistently show an improvement of spirometric values, yet the subjects continue to report decreased dyspnea and improved scores on their SGRQ. It is hypothesized that these symptomatic benefits are the result of physiologic changes other than lung compliance, especially since many studies report incomplete atelectasis of the intervened lobe (even with the lobar exclusion approach as the goal) [65, 76]. Ventilation/perfusion (V/Q) scintigraphy was performed following unilateral placement of EBVs in 6 COPD patients who had destruction of their left upper lobe [77]. By 90-day followup, there was a significant decrease in the ventilation and perfusion of the targeted left upper lobe (*P* = .01 and  .02) though only one of the 6 achieved atelectasis of the targeted region. Furthermore, the subjects had an increase in the ventilation and perfusion of the contralateral lung [77]. Placement of the valve leads to reduced ventilation, while concomitant decreased perfusion may be the result of subsequent hypoxic vasoconstriction of the same region [77]. Furthermore, the beneficial effect secondary to a reduction in physiologic dead space leads to more efficient ventilation, and the reduced hyperinflation may divert airflow to less obstructive airways [76]. 

Currently endobronchial valves are not approved by the FDA for the use in patients with emphysema. Larger, randomized controlled trials are currently underway to better assess the role they may play as a minimally invasive alternative therapy in LVR.

### 4.6. Biologic Lung Volume Reduction (Sealants)

The use of a fibrin-based glue to collapse, scar, and seal off target regions of abnormal lung was first described in sheep [78]. The sealant occludes the airway distally, causes resorption atelectasis, and subsequent airspace inflammation followed by remodeling and scarring [65]. An open-label phase 1 trial deemed the use of the sealant in COPD patients safe [79]. The sealant was placed in either two or four unilateral pulmonary segments in 6 men with heterogeneous emphysema. There were no serious complications and all patients were discharged on posttreatment day 1 [79]. 

Since then, three phase-two trials have been summarized [80]. Fifty patients were divided into two treatment groups (28 in the 10 mL of sealant group and 22 in the 20 mL of sealant group). A flexible bronchoscope was used to advance into the selected subsegmental orifice and wedged in position. A dual lumen catheter was advanced into the airways with the tip stationed 3-4 cm distal to the bronchoscope. The BioLVR fibrinogen and thrombin solutions were administered through the catheter over 10–15 seconds. Immediately afterward, 60 mL of air was injected through the working channel of the bronchoscope to advance the reagent distally. After 30 seconds, the bronchoscope was then moved to the next intervention site [80]. 

No deaths were seen, but 4 serious adverse events were documented. These consisted of pneumonia, aspiration pneumonia followed by myocardial infarct, pleuritic chest pain with subsequent fall related to analgesia use and pulmonary embolism [80]. Forty-two patients experienced leukocytosis, fever, and malaise, an expected side effect due to the inflammation that ensues from the procedure. The primary endpoint of a significant reduction in RV/TLC at 3 months was achieved in both dosing groups. At 6-months, only the FEV_1_ remained significantly improved compared to baseline in the lower dose treatment group. However, significant improvements were sustained in all physiological outcomes in the higher dose treatment group compared to baseline [80]. The future of biologic or nonsurgical lung volume reduction needs further analysis by larger randomized controlled trials with appropriate sham procedure.

## 5. Conclusion

While largely preventable, COPD is a progressive chronic lung disorder with systemic consequences ranging from respiratory constraints, muscle wasting, chronic inflammation, repeat infections and social limitations including isolation and depression. 

Current pharmacotherapy has been able to decrease the number of exacerbations but remains inadequate at halting disease progression. Development and clinical testing of novel therapies are thus imperative in order to make strides in this field. This review of the state-of-the-art and novel pharmacotherapy and experimental minimally invasive interventions underscore the advances that have been made in the past decade with regards to the amelioration of symptoms and exercise limitations of COPD. Further clinical studies are needed to bring the promise of improved treatment and halting disease progression to fruition.

## Figures and Tables

**Table 1:** Spirometric classification of COPD severity: gold staging criteria.

Stage I: mild	FEV_1_/FVC < 0.70
	FEV_1_ ≥ 80% predicted
Stage II: moderate	FEV_1_/FVC < 0.70
	50% ≤ FEV_1_ < 80% predicted
Stage III: severe	FEV_1_/FVC < 0.70
	30% ≤ FEV_1_ < 50% predicted
Stage IV: very severe	FEV_1_/FVC < 0.70
	FEV_1_ < 30% predicted or FEV_1_ < 50% predicted plus chronic respiratory failure

FEV_1_: forced expiratory volume in the first second (postbronchodilator).

FVC: forced vital capacity.

Respiratory failure: arterial partial pressure of oxygen (PaO_2_) less than 8.0 kPa (60 mm Hg) with or without arterial partial pressure of CO_2_ (PaCO_2_) greater than 6.7 kPa (50 mm Hg) while breathing air at sea level.

Adapted from http://www.goldcopd.com/, updated 2009.
